# Toll-like Receptor 2 is Involved in Calcium Influx and Acrosome Reaction to Facilitate Sperm Penetration to Oocytes During *in vitro* Fertilization in Cattle

**DOI:** 10.3389/fcell.2022.810961

**Published:** 2022-02-24

**Authors:** Dongxue Ma, Mohamed Ali Marey, Masayuki Shimada, Akio Miyamoto

**Affiliations:** ^1^ Global Agromedicine Research Center (GAMRC), Obihiro University of Agriculture and Veterinary Medicine, Obihiro, Japan; ^2^ Department of Theriogenology, Faculty of Veterinary Medicine, Damanhur University, Behera, Egypt; ^3^ Graduate School of Integrated Sciences for Life, Hiroshima University, Higashi-Hiroshima, Japan

**Keywords:** sperm, toll-like receptor 2, *in vitro* fertilization, acrosome reaction, intracellular Ca^2+^ influx, bovine

## Abstract

Cumulus cells of ovulated cumulus-oocyte complexes (COCs) express Toll-like receptor 2 (TLR2), pathogen recognition receptors, to recognize and react to sperm signals during fertilization. Sperm also express TLR2, but its contribution to the sperm-oocytes crosstalk is still unclear. Here, we adapted the *in vitro* fertilization (IVF) model to characterize the potential relevance of sperm TLR2 in sperm-oocytes interactions during fertilization in bovine. The IVF results showed that the ligation of sperm TLR2 with its specific antagonist/agonist resulted in down/up-regulation of the cleavage and blastocyst rates either in COCs or cumulus-free oocytes, but not in zona pellucida (ZP)-free oocytes. The computer-assisted sperm analysis (CASA) system revealed that sperm motility parameters were not affected in TLR2 antagonist/agonist-treated sperm. However, fluorescence imaging of sperm-ZP interactions revealed that the blockage or activation of the TLR2 system in sperm reduced or enhanced both binding and penetration abilities of sperm to ZP compared to control, respectively. Flow cytometrical analysis of acrosome reaction (AR) demonstrated that the TLR2 system adjusted the occurrence of AR in ZP-attached sperm, suggesting that sperm TLR2 plays physiological impacts on the sperm-oocyte crosstalk *via* regulating ZP-triggered AR in sperm. Given that calcium (Ca^2+^) influx is a pre-requisite step for the induction of AR, we investigated the impact of the TLR2 system on the ionophore A23187-induced Ca^2+^ influx into sperm. Notably, the exposure of sperm to TLR2 antagonist/agonist reduced/increased the intracellular Ca^2+^ level in sperm. Together, these findings shed new light that the TLR2 system is involved in sperm AR induction which enables sperm to penetrate and fertilize oocytes during the fertilization, at least *in vitro*, in cows. This suggests that sperm possibly developed a quite flexible sensing mechanism simultaneously against pathogens as well as COCs toward fertilization with the same TLR2 of the innate immune system.

## Introduction

Fertilization failure is one of the main reasons for infertility which has been attributed to either sperm or oocyte factors (Kashir et al., 2010). Male factors represent approximately 40% of all infertility cases in humans (Kumar and Singh., 2015), such as abnormal sperm parameters which are usually associated with virus/bacterial infection and inflammation. In mammals, sperm are not capable of fertilizing oocytes immediately after ejaculation, but they must first undergo a period of preparation including capacitation and acrosome reaction (AR) (Liu et al., 2012).

Sperm capacitation is a prerequisite step to successful fertilization (Austin, 1951; Chang, 1951) that involves a series of biochemical transformations, including changes in sperm metabolism, intracellular pH, intracellular cyclic adenosine monophosphate (cAMP), and intracellular calcium concentration, all of which prepare sperm to undergo AR to penetrate the zona pellucida (ZP) and fertilize oocytes (Breitbart et al., 2005; Kwon et al., 2013). Despite investigations, it is not completely understood whether fertilizing spermatozoon initiates its AR during its voyage through the cumulus cells or when it binds to the ZP (Gadella, 2012). However, fertilizing spermatozoon must undergo AR to penetrate the ZP, in which the outer acrosomal membrane fuses with the overlying plasma membrane (Yanagimachi, 1994; Honda et al., 2002; Mao and Yang, 2013). Therefore, only the acrosome-reacted sperm are existent in the perivitelline space and can fuse with the plasma membrane of the oocyte to effect fertilization (Austin, 1975; Saling et al., 1979; Toshimori, 1982; Inoue et al., 2005; Avella and Dean., 2011).

The regulation of calcium (Ca^2+^) influx plays a key role in many physiological cell functions, such as cell growth, apoptosis, exocytosis, muscle contraction, and gene transcription (Chun and Prince, 2006). In sperm, it is well known that Ca^2+^ release and influx are essential for the acquisition of sperm fertilizing competence through the induction of capacitation and AR. The *in vitro* capacitation of bovine sperm with heparin is accomplished by the extracellular Ca^2+^ uptake by the sperm through Ca^2+^ channels which ultimately increase the intracellular Ca^2+^ levels in the sperm head leading to activation of adenylyl cyclase to form cAMP, which further activates protein kinase A (PKA) to phosphorylate protein tyrosine (Parrish et al., 1999; Marquez and Suarez 2004; Ijiri et al., 2012). Although the specific sequence of events that trigger the AR after capacitation is not fully understood, there is evidence that it involves elevations of intracellular pH, cytosolic Ca^2+^, membrane hyperpolarization, acrosomal alkalization, and acrosomal Ca^2+^ release (Darszon et al., 2011; Nishigaki et al., 2014; Stival et al., 2016; Chávez et al., 2017). These events promote acrosome swelling, deformation of the outer acrosomal membrane (OAM), interaction and docking with the overlying plasma membrane (PM), and finally, the fusion between OAM and PM that promotes acrosomal exocytosis and release of hydrolytic enzymes, principally, the trypsin-like acrosin to penetrate the ZP and fertilize the oocyte (Mayorga et al., 2007; Sosa et al., 2014; Aldana et al., 2021).

Toll-like receptors (TLRs) are innate immune cell receptors that specifically recognize pathogenic microorganisms and mount an early immune response, resulting in the production of pro-inflammatory mediators (Akira et al., 2001). Moreover, TLRs are involved in several reproductive functions including ovulation, fertilization, gestation, and parturition (Kannaki et al., 2011). In males, TLRs play a role in steroidogenesis and spermatogenesis (Saeidi et al., 2014). Among TLRs family members, TLR2 and TLR4 are expressed in cumulus cells of cumulus-oocyte complexes (COCs) and play immuno-protective functions critical for cell survival during ovulation and fertilization (Shimada et al., 2006; Liu et al., 2008).

Specifically, it has been shown that co-culturing of sperm and COCs during *in vitro* fertilization (IVF) induces activation of cumulus cells TLR2/4 signaling pathway in mice (Shimada et al., 2008). In that model, the release of hyaluronidase from sperm degrades the hyaluronan-rich matrix of the COCs releasing hyaluronan fragments which subsequently act as endogenous ligands for cumulus cells TLR2/4. This binding could activate the production of certain cytokines/chemokines essential for sperm capacitation, thus enhance fertilization. The findings suggest a physiological role of the TLR2/4 pathway as a regulatory loop between sperm and COCs during fertilization (Stern et al., 2006; Shimada et al., 2008). Of note, human sperm express TLR2, which is functional for the recognition of bacterial endotoxins during infection (Fujita et al., 2011), but their role in sperm interactions with oocytes during fertilization is still unknown. Most recently, we demonstrated that TLR2 is localized in the posterior segment of the bull sperm head (Akthar et al., 2020). Therefore, we adapted the IVF model to clarify the potential relevance of sperm TLR2 in sperm-oocytes interactions during fertilization in bovine. Our initial observations prompted us to hypothesize that bull sperm TLR2 is functional in sperm-oocyte interactions during fertilization. To examine this hypothesis, sperm TLR2 was ligated by their specific antagonist/agonist before being co-cultured with oocytes. Then, we investigated the embryo cleavage and developmental competence, sperm-ZP binding and penetration rates, and ZP or ionophore A23187-induced AR. Further, the direct impact of sperm TLR2 blockage/activation on sperm motility kinetics, AR, and intracellular Ca^2+^ influx was analyzed.

## Materials and Methods

### Ethical Approval

Animal experiments described in this article were conducted following the Guiding Principles for the Care and Use of Research Animals Promulgated by Obihiro University of Agriculture and Veterinary Medicine, Japan. The protocol was approved by the Committee on the Ethics of Animal Experiments of the Obihiro University of Agriculture and Veterinary Medicine (Permit number 19–111).

### Experimental Design

**FIGURE 1:**
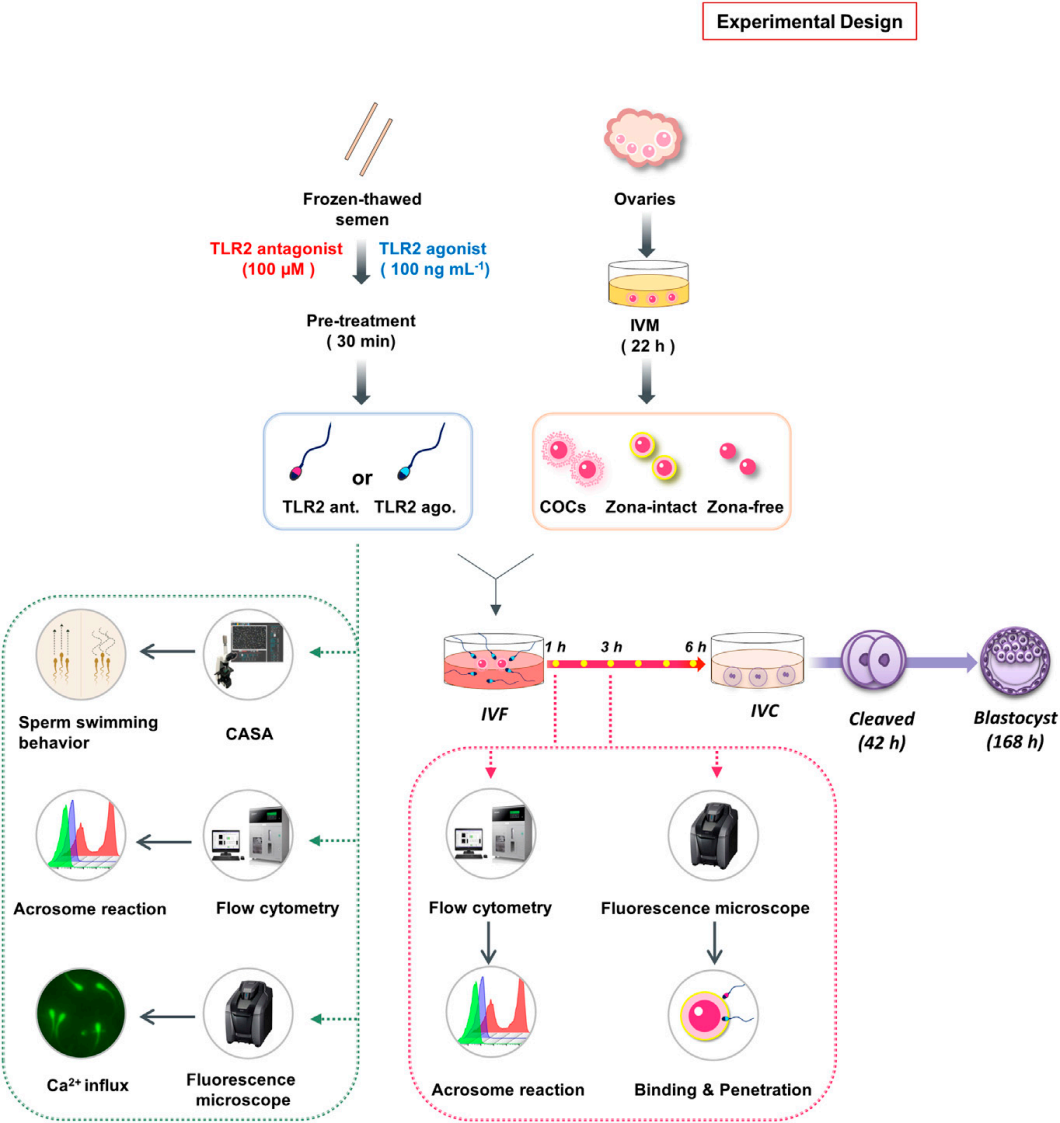
Schematic representation of the study experimental design. Initially, to investigate the potential contribution of sperm TLR2 in sperm-oocyte interaction during *in vitro* fertilization, TLR2 antagonist (CU-CPT22; 100 µM) or TLR2 agonist (Pam3Cys; 100 ng ml^−1^) was used for the blockage or activation of TLR2 in frozen-thawed bull sperm for 30 min, respectively. Sperm were then washed, adjusted to a concentration of 0.5 × 10^6^ sperm ml^−1^, and co-cultured with matured intact COCs, cumulus-free (zona intact), or zona-free oocytes for 6 h. Cleavage rate and blastocyst rate were evaluated at 42 and 168 h post-insemination respectively. Moreover, the ability of TLR2 antagonist/agonist treated sperm to bind or penetrate ZP and the subsequent induction of AR was evaluated after 1 and 3 h of the co-culture using fluorescence microscopy and flow cytometry, respectively. Based on the results, more focus was given to the direct impact of blockage/activation of sperm TLR2 on sperm motility parameters, AR, and intracellular Ca^2+^ influx using CASA analysis, flow cytometry, and fluorescence microscopy, respectively.

The design and framework of multiple investigations performed in the present study was illustrated in Figure 1. Initially, to investigate the potential contribution of sperm TLR2 in sperm-oocyte interaction during *in vitro* fertilization, a specific TLR2 antagonist (CU-CPT22) or TLR2 agonist (Pam3Cys) was used for the blockage or activation of sperm TLR2, respectively. It was shown that cumulus cells of COCs also express TLR2 and their activation or blockage modulates the fertilization ratio (Shimada et al., 2008). To investigate the impact of “sperm TLR2” on fertilization, the sperm were basically pre-exposed to TLR2 antagonist or agonist before their co-culture with oocytes under IVF conditions. Washed frozen-thawed bull sperm were pre-exposed either to TLR2 antagonist (100 µM) or TLR2 agonist (100 ng ml^−1^) for 30 min, washed, adjusted to a concentration of 0.5×10^6^ sperm ml^−1^, and co-cultured with matured COCs for 6 h. Then, cleavage rate and blastocyst rate were evaluated at 42 and 168 h post-insemination, respectively. Our initial observations showed that the blockage/activation of sperm TLR2 inhibited/stimulated the oocyte fertilization and embryo developmental competence. To understand the underlying mechanisms, we assessed the impact of sperm TLR2 on the sperm-cumulus cells interaction or sperm-ZP fusion *via* co-culturing of TLR2 antagonist/agonist treated sperm with matured cumulus-free (zona-intact) or zona-free oocytes for 6 h, respectively and the cleavage rate was determined at 42 h post-insemination. Moreover, the ability of TLR2 antagonist/agonist treated sperm to bind and/or penetrate ZP and the subsequent induction of AR was evaluated after 1 and 3 h of the co-culture using fluorescence microscopy and flow cytometry, respectively. Based on the results, more focus was given to the direct impact of TLR2 on sperm motility parameters, AR, and intracellular Ca^2+^ influx using CASA analysis, flow cytometry, and fluorescence microscopy, respectively.

### Cumulus-Oocyte Complexes (COCs) Collection and *in vitro* Maturation (IVM)

Bovine ovaries were collected from the local slaughterhouse (Obihiro, Hokkaido, Japan), kept in saline supplemented with 1% penicillin-streptomycin (Gibco, Grand Island, United States) at approximately 35°C, and immediately transported to the laboratory within 1–2 h. COCs collection and IVM were performed as previously described (Ideta et al., 2013) with minor modifications. Briefly, COCs were aspirated from individual visible antral follicles of 2–6 mm in diameter using a 10 ml syringe attached to an 18 G needle. Then, oocytes were washed 3 times in oocyte collection medium (OCM; Research Institute for the Functional Peptides Co., Ltd.) supplemented with 10% fetal bovine serum (FBS, Biowest). Each 40–50 oocytes with a homogenous cytoplasm and surrounded by at least three layers of compact cumulus cells were matured *in vitro* using 500 μl of high performance-modified 199 medium (HP-M199; Research Institute for the Functional Peptides Co., Ltd.) supplemented with 10 ng ml^−1^ epidermal growth factor (EGF, Sigma) and 10% FBS for 22 h at 38.5°C in 5% CO_2_ with humidified air.

### Blockage and Activation of TLR2 Pathway in Sperm

A synthetic TLR2 antagonist (CU-CPT22, Calbiochem) (Cheng et al., 2012; Grabowski, et al., 2020) or Pam3Cys, a synthetic TLR2 ligand (ab142085, Abcam) (Reitermann et al., 1989) was used to specifically and selectively block or activate the sperm TLR2, respectively. Initially, preliminary dose-dependent and time-dependent experiments were conducted to identify cytotoxic or detrimental effects of TLR2 antagonist and agonist on sperm motility and viability as analyzed by CASA and flow cytometry (Supplementary Table S1 and Supplementary Figure S1). Next, based on our previous investigations (Ezz et al., 2019; Akthar et al., 2019; Morillo, et al., 2020; Elesh et al., 2021) and our initial trials, the pre-treatment of sperm by TLR2 antagonist (100 µM) or agonist (100 ng ml^−1^) for 30 min was designated as an effective model to test our hypothesis without generating any cytotoxic or detrimental effects on sperm motility and viability.

Frozen semen straws were obtained from three highly fertile Holstein bulls from the Genetics Hokkaido Association (Hokkaido, Japan). Semen straws were thawed at 37 °C for 30 s, pooled together, and washed twice using a modified sperm-tyrode’s albumin lactate pyruvate (SP-TALP) (Parrish et al., 1988; Marquez and Suarez, 2007) at 300 g for 5 min at room temperature. After washing, sperm were treated either by TLR2 antagonist (100 µM) or agonist (100 ng ml^−1^) for 30 min, respectively. TLR2 antagonist (Stock solution: 100 mM) was dissolved in DMSO and used at a concentration of 100 μM (100 μM = 36.24 μg ml^−1^) while TLR2 agonist (Stock solution: 100 μg ml^−1^) was dissolved in 50% ethanol and used at a concentration of 100 ng ml^−1^. Similarly, the sperm group with 0.1% DMSO or 0.05% ethanol was kept as a control group for TLR2 antagonist- or TLR2-agonist treated sperm, respectively. Sperm were then washed twice at 300 g for 5 min in SP-TALP and resuspended in fertilization medium IVF100 (Research Institute for the Functional Peptides, Yamagata, Japan).

### *In vitro* Fertilization (IVF)

After maturation, COCs were collected and either kept intact, or cumulus cells and/or zona pellucida were removed. Cumulus cells were removed by gentle repeated pipetting with 300 μg ml^−1^ hyaluronidase (Sigma-Aldrich, Steinheim, Germany). The preparation of zona-free oocytes was performed as previously described (Takahashi et al., 2013) with slight modifications. Briefly, the zona pellucida of denuded oocytes were dissolved with 5 mg ml^−1^ pronase (protease from *Streptomyces* griseus, Sigma-Aldrich, Steinheim, Germany) for 1 min under visual monitoring. The thin residual layers were physically removed using a narrow bore glass pipette. Zona-free oocytes were rapidly washed 5 times with 0.1% bovine serum albumin (BSA) in PBS and kept at 38.5°C under 5% CO_2_ in humidified air for 1 h recovery. IVF was achieved by co-culturing of TLR2 antagonist/agonist-treated sperm (0.5 ×10^6^ sperm ml^−1^) either with intact COCs, cumulus-free oocytes, or zona-free oocytes (10–15 oocytes) in 100 µL droplets of IVF100 medium under mineral oil for 6 h at 38.5 °C in 5% CO_2_ in humidified air.

### *In vitro* Culture (IVC)

After IVF, cumulus cells were removed by repeated pipetting with 300 μg ml^−1^ hyaluronidase. Presumptive zygotes (n = 30–35) were transferred to 400 µL BO-IVC medium (IVF-bioscience, Poland, Sokolow Podlaski) in 4-well plates under mineral oil at 38.5°C in a humidified atmosphere of 5% O_2_, 5% CO_2_, and 90% N_2_. Cleavage rate and blastocyst development were assessed at 42 and 168 h (Day 7) post-insemination, respectively (Day 0 = day of fertilization).

### Sperm-ZP Binding and Penetration Assay

For investigating the sperm-ZP binding and penetration, TLR2 antagonist/agonist-treated sperm (0.5×10^6^ sperm ml^−1^) were co-incubated with cumulus-free oocytes (n = 15–20) in each 100 µL fertilization droplets at 38.5°C in 5% CO_2_ in humidified air for 1 and 3 h. At 1 h and 3 h of co-incubation, sperm-oocyte complexes were washed 10 times in PBS containing 1 mg ml^−1^ polyvinyl alcohol (PVA-PBS) to remove ZP-loosely attached sperm while only ZP-strongly attached sperm were counted as a sperm-ZP binding ratio (Takahashi et al., 2013). For assessment of sperm-ZP penetration, sperm-oocyte complexes were repeatedly aspirated with a pipette of an inner diameter slightly smaller than the size of the oocyte to remove all attached sperm and only those with heads embedded in the ZP or perivitelline space were counted as sperm-ZP penetration ratio (Liu et al., 2004). Next, oocytes were fixed in 5% glutaraldehyde solution (072-02262, Fujifilm) in PBS for 30 min at room temperature then, washed with PVA-PBS before being incubated with 5 μg ml^−1^ Hoechst 33342 (Sigma-Aldrich, B2261) for 15 min in the dark (Grullón et al., 2013). Finally, oocytes were placed in droplets of glycerol, mounted on the slide, and covered with a cover slide. The number of sperms bound or penetrated the ZP of each oocyte was counted independently by 2 observers using a fluorescence microscope (Keyence, BZ-X800, Osaka, Japan).

### Assessment of Sperm Kinematics

The impact of pre-treatment of sperm by TLR2 antagonist/agonist on sperm motility parameters was analyzed using the computer-assisted sperm analysis system (CASA) (SMAS; Kaga Electronics, Tokyo, Japan). Sperm were incubated in SP-TALP medium supplemented either by TLR2 antagonist (100 μM) or TLR2 agonist (100 ng ml^−1^), for 30 min, washed twice, resuspended in SP-TALP at a concentration 5–10×10^6^ sperm ml^−1^, and cultured for further 1 or 3 h. Sperm motility parameters (Total motility (%), progressive motility (%), average path velocity (VAP, µm/s), straight-line velocity (VSL, µm/s), curvilinear velocity (VCL, µm/s), amplitude of lateral head displacement (ALH, µm), beat cross frequency (BCF, Hz), straightness (STR, %), and linearity (LIN, %)), were assessed at different time points (0, 1, and 3 h) using CASA system. CASA was performed based on a previous method with minor modifications (Kanno et al., 2017). Briefly, a 3 μl of the sperm sample was pipetted and loaded into a pre-warmed (37°C) standard count four chamber Leja slide (SC 20-01-04-B). To analyze sperm motility parameters, a minimum of 200 sperm at three different fields were examined in each group.

### Assessment of Acrosome Reaction (AR)

Sperm AR can be induced either by ZP, as a physiological stimulus, during sperm-ZP binding or by calcium ionophore as a chemical stimulus (Yanagimachi, 1994; Strünker et al., 2011). To investigate the impact of pre-treatment of sperm by TLR2 antagonist/agonist on the ZP-inducted AR, TLR2 antagonist/agonist pre-treated sperm were co-cultured with cumulus-free oocytes for 1 h or 3 h under IVF conditions. Then, ZP-bound sperm were collected, as mentioned above, and incubated with 25 μg ml^−1^ fluorescein peanut agglutinin FITC-conjugate (PNA-FITC; Vector Laboratories, FL-1071) for 8 min at 38.5°C in dark. The percent of acrosome-reacted sperm was analyzed by flow cytometry (Sony SH800 Cell Sorter, Tokyo, Japan). For chemical induction of AR by calcium ionophore, motile sperm were prepared using a modified swim-up method (Parrish et al., 1988) and treated by TLR2 antagonist/agonist as previously described. Then, sperm were stimulated with or without calcium ionophore A23187 (1 μM, C7522, Sigma) for 60 min at 38.5°C in the dark. After incubation, sperm were washed twice and double-stained with 1 μl of 1 mg ml^−1^ propidium iodide (cell viability test *via* detection of plasma membrane damage (PMD)) (PI; P4170, Sigma) and 1 μl of 5 mg ml^−1^ PNA-FITC mixed with 200 μl sperm dilution and incubated for 8 min at 38.5°C in dark. The percentage of PI-negative and PNA-FITC-positive (live and acrosome reacted) sperm were determined immediately by flow cytometry. Additionally, stained sperm samples were smeared, fixed, and examined under fluorescence microscope.

### Single-Cell Imaging Measurement of Intracellular Calcium Influx

Assessment of intracellular calcium influx was recorded during the A23817-induced AR assay as previously described (Marquez and Suarez, 2007) with minor modifications. Immediately before the addition of A23817 to induce AR as above-mentioned, each sample was loaded with 5 µM Fluo-4 AM (F311, Dojindo, Japan) for 40 min at 38.5°C in the dark and washed twice to remove free Fluo-4 AM. Then, Fluo-4 AM-loaded sperm were incubated for another 20 min with a fresh medium before adding calcium ionophore A23187 (1 µM as a final concentration). The sperm fluorescence was measured by the fluorescence microscope. Images were captured every 5 min for a total of 1 h, with A23187 added after the initial five readings (every 1 min). The images were analyzed using BZ-X800 Analyzer and each sperm head was selected as the region of interest. Data were normalized using the following equation (F/F0)-1. where F0 is the average of the first five readings before the addition of A23187 and F is the fluorescence intensity obtained at each time point.

### Statistical Analysis

Data are presented as the mean ± S.E.M of 3−5 independent experiments. Statistical analyses were performed using GraphPad Prism five software (GraphPad Software, La Jolla, CA, United States). One-way analysis of variance (ANOVA) followed by Bonferroni’s post-comparison test (>two groups) or two sample *t*-test (two groups) was used to compare the mean differences among the groups. Data were considered to be statistically significant at (*p < 0.05, **p < 0.01, ***p < 0.001, or ****p < 0.0001)*.*

## Results

### Blockage/Activation of Sperm TLR2 Suppressed/Enhanced the Cleavage and Blastocyst Rates in COCs

**FIGURE 2:**
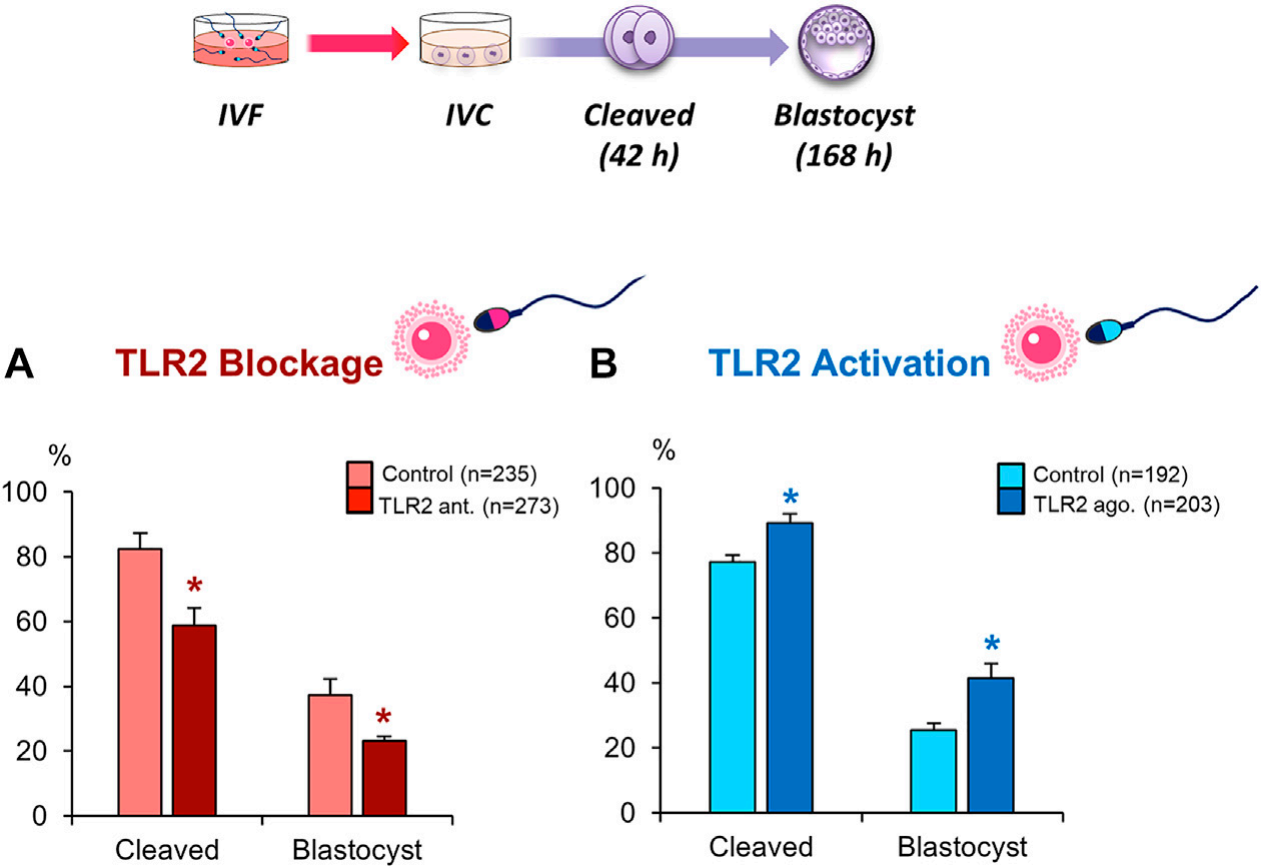
Blockage/activation of sperm TLR2 suppressed/enhanced the cleavage and blastocyst rates in COCs. Sperm were pre-treated by **(A)** TLR2 antagonist (100 µM) or **(B)** TLR2 agonist (100 ng ml^−1^) for 30 min, washed, and co-cultured with cumulus-free(zona-intact) and zona-free oocytes. Percentage of fertilized oocytes observed after 42 h post insemination and blastocyst on Day 7 (Day 0 = day of fertilization). Data reported as means ± S.E.M. Different superscript asterisks denote a significant difference (*p <* 0.05). The number of presumptive zygotes for each treatment group (from three independent experiments) is specified above each Figure.

To investigate the potential role of sperm TLR2 in sperm-oocyte interaction, sperm were pre-treated by TLR2 antagonist/agonist for 30 min, washed, and further co-cultured with intact COCs for 6 h under IVF conditions. The results showed that the blockage of sperm with TLR2 antagonist induced a substantial decline in the cleavage and blastocyst rates (Figure 2A). Meanwhile, the activation of sperm with TLR2 agonist enhanced cleavage and blastocyst rates (Figure 2B).

### Blockage/Activation of Sperm TLR2 Suppressed/Enhanced the Cleavage Rate in Cumulus-free Oocytes, but Not in Zona-free Oocytes

**FIGURE 3:**
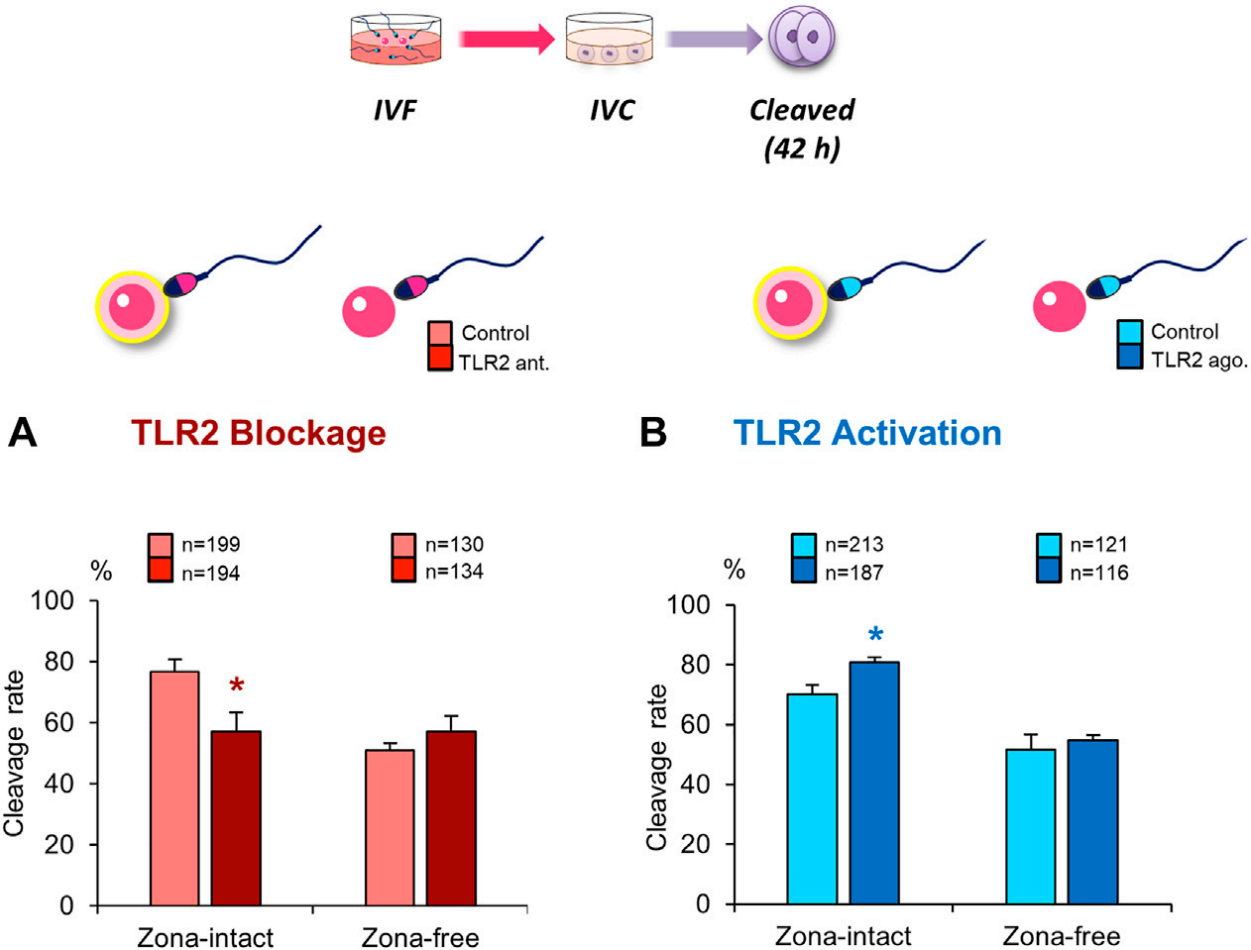
Blockage/activation of sperm TLR2 suppressed/enhanced the cleavage rate in cumulus-free oocytes, but not in zona-free oocytes. Sperm were pre-treated by **(A)** TLR2 antagonist (100 µM) or **(B)** TLR2 agonist (100 ng ml^−1^) for 30 min, washed, and co-cultured with cumulus-free (zona-intact) and zona-free oocytes. Percentage of fertilized oocytes observed after 42 h post insemination. Data reported as means ± S.E.M. Different superscript asterisks denote a significant difference (*p <* 0.05). The number of presumptive zygotes for each treatment group (from three independent experiments) is specified above each Figure.

To further explore the impact of sperm TLR2 on sperm crosstalk with cumulus cells and/or ZP, TLR2 antagonist/agonist treated sperm were co-cultured with matured cumulus-free or zona-free oocytes for 6 h, and cleavage rate was analyzed. Likewise, results revealed that the blockage/activation of sperm TLR2 suppressed/increased the cleavage rate in cumulus-free oocytes, but not with zona-free oocytes, compared to control (Figures 3A,B). These findings could imply that sperm TLR2 plays a pivotal role in sperm-ZP interactions.

### TLR2 Pathway Mediates Sperm-ZP Binding and Sperm-ZP Penetration Abilities

**FIGURE 4:**
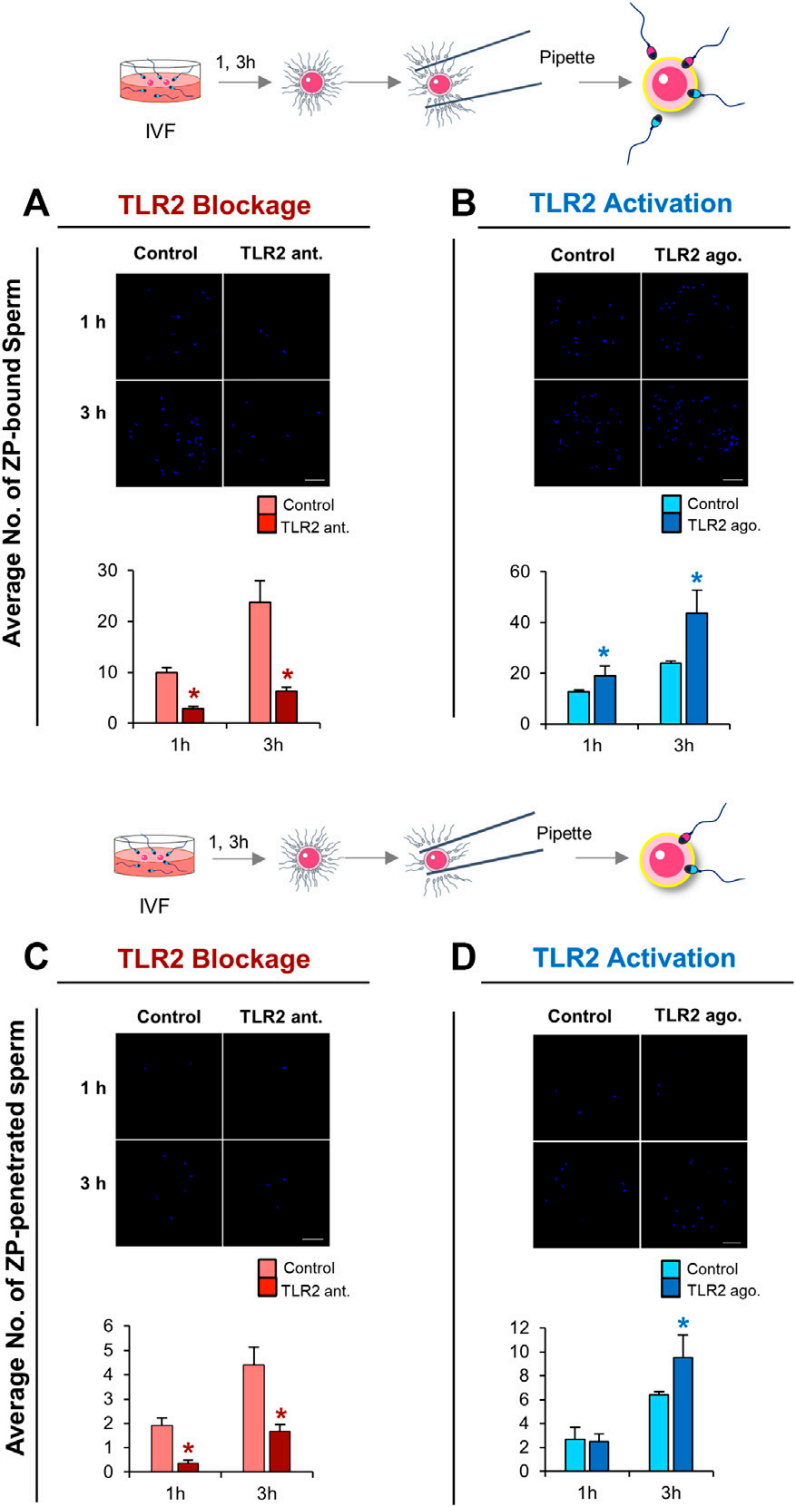
TLR2 pathway mediates sperm-ZP binding and sperm-ZP penetration abilities. Sperm were pre-treated by TLR2 antagonist (100 µM) or TLR2 agonist (100 ng ml^−1^) for 30 min, washed, and co-cultured with cumulus-free oocytes (n = 15–20) for 1 and 3 h. Sperm-oocyte complexes were washed to remove ZP-loosely attached sperm while only ZP-strongly attached sperm were counted as sperm-ZP binding ratio after staining with Hoechst 33342 and observed at ×200 magnification. Sperm with heads embedded in the ZP or perivitelline space were counted as sperm-ZP penetration ratio **(A)** Representative image of zona-binding observed in control and TLR2 antagonist treated group and the average number of sperm bound to the ZP at 1 and 3 h was counted **(B)** Representative image of zona-binding observed in control and TLR2 agonist treated group and the average number of sperm bound to the ZP at 1 and 3 h was counted **(C)** Representative image of zona-penetration observed in control and TLR2 antagonist treated group and the average number of sperm penetrated to the ZP at 1 and 3 h was counted **(D)** Representative image of zona-penetration observed in control and TLR2 agonist treated group and the average number of sperm penetration to the ZP at 1 and 3 h was counted. Data reported as means ± S.E.M of three independent experiments. Different superscript asterisks denote a significant difference (*p* < 0.05). Scale bar = 50 μm.

Based on the above-mentioned results, we hypothesized that the activation of sperm TLR2 is a pre-requisite step for enabling sperm to bind and/or penetrate ZP. To test this hypothesis, the ability of TLR2 antagonist/agonist treated sperm to bind and/or penetrate ZP was tested at 1 and 3 h of co-incubation with cumulus-free oocytes. The results showed that the blockage/activation of sperm TLR2 suppressed/increased the number of ZP-bound sperm at 1 and 3 h of co-culture (Figures 4A,B). Likewise, pre-treatment of sperm with TLR2 antagonist suppressed the average number of ZP-penetrated sperm compared to the control group at 1 and 3 h (*p* < 0.05) (Figure 4C). Conversely, pre-treatment of sperm with TLR2 agonist increased the average number of ZP-penetrated sperm compared to the control group at 3 h (*p* < 0.05) (Figure 4D).

### Pre-treatment of Sperm With TLR2 Antagonist/Agonist did Not Disturb Sperm Motility Parameters

To determine whether the impact of sperm TLR2 on sperm-oocyte communication was attributed to their direct effect on sperm motility parameters, we analyzed different motility parameters of TLR2 antagonist/agonist pre-treated sperm at different time points (0, 1, and 3 h) of the next culture using CASA system. The results showed that the pre-treatment of sperm with TLR2 antagonist/agonist did not affect all sperm motility parameters, related to their fertilizing competence (Total motility (%), progressive motility (%), average path velocity (VAP, µm/s), straight-line velocity (VSL, µm/s), curvilinear velocity (VCL, µm/s), the amplitude of lateral head displacement (ALH, µm), beat cross frequency (BCF, Hz), straightness (STR, %), and linearity (LIN, %)), at all tested time points (Supplementary Table S2A,B **).** These findings prompted us to hypothesize that TLR2 regulates sperm interactions with oocytes without interfering with sperm motility parameters.

### TLR2 System Impacts the ZP-Induced Acrosome Reaction in Sperm Under IVF Conditions

**FIGURE 5:**
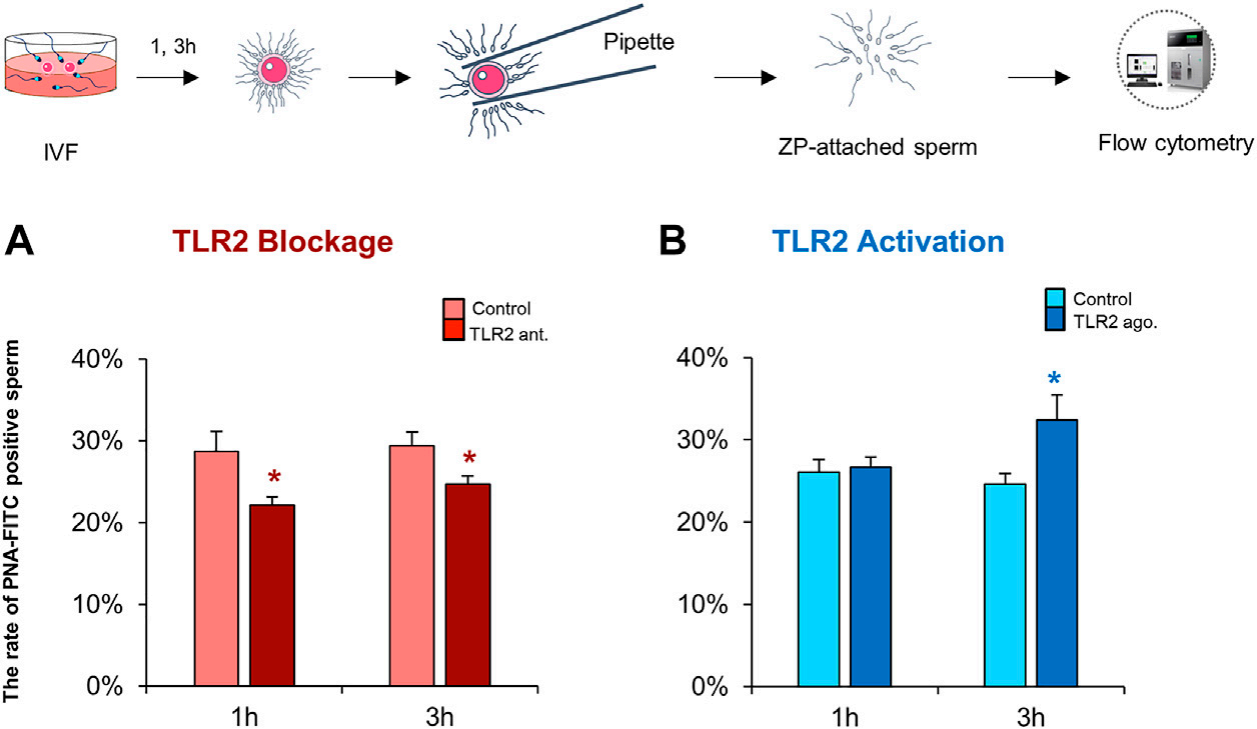
TLR2 system impacts the ZP-induced acrosome reaction in sperm under IVF conditions. Sperm were pre-treated by TLR2 antagonist (100 µM) or TLR2 agonist (100 ng ml^−1^) for 30 min, washed, and co-cultured with cumulus-free oocytes for 1 and 3 h. Then, ZP-attached sperm were collected and incubated with 25 μg ml^−1^ fluorescein peanut agglutinin FITC-conjugate (PNA-FITC) for 8 min at 38.5°C in dark. The percent of acrosome-reacted sperm was analyzed by flow cytometry **(A)** TLR2 antagonist reduces the ZP-induced acrosome reaction in sperm under IVF conditions **(B)** TLR2 agonist increases the ZP-induced acrosome reaction in sperm under IVF conditions. Data reported as means ± S.E.M of five independent experiments. Different superscript asterisks denote a significant difference (*p* < 0.05).

Induction of AR is an essential step for mammalian sperm to penetrate the ZP and fertilize oocytes (Breitbart et al., 2005; Kwon et al., 2013). The results of flow cytometry revealed that the blockage of sperm TLR2 suppressed the induction of AR in ZP-bound sperm at 1 and 3 h under IVF conditions compared to control (Figure 5A). While the activation of sperm TLR2 increased the induction of AR in ZP-bound sperm at 3 h under IVF conditions (Figure 5B). These results may account for the ability of sperm TLR2 to regulate sperm-ZP binding and penetration and thereby fertilizing oocytes *via* the induction of AR.

### TLR2 System Impacts A23187-Triggered Acrosome Reaction in Sperm

**FIGURE 6:**
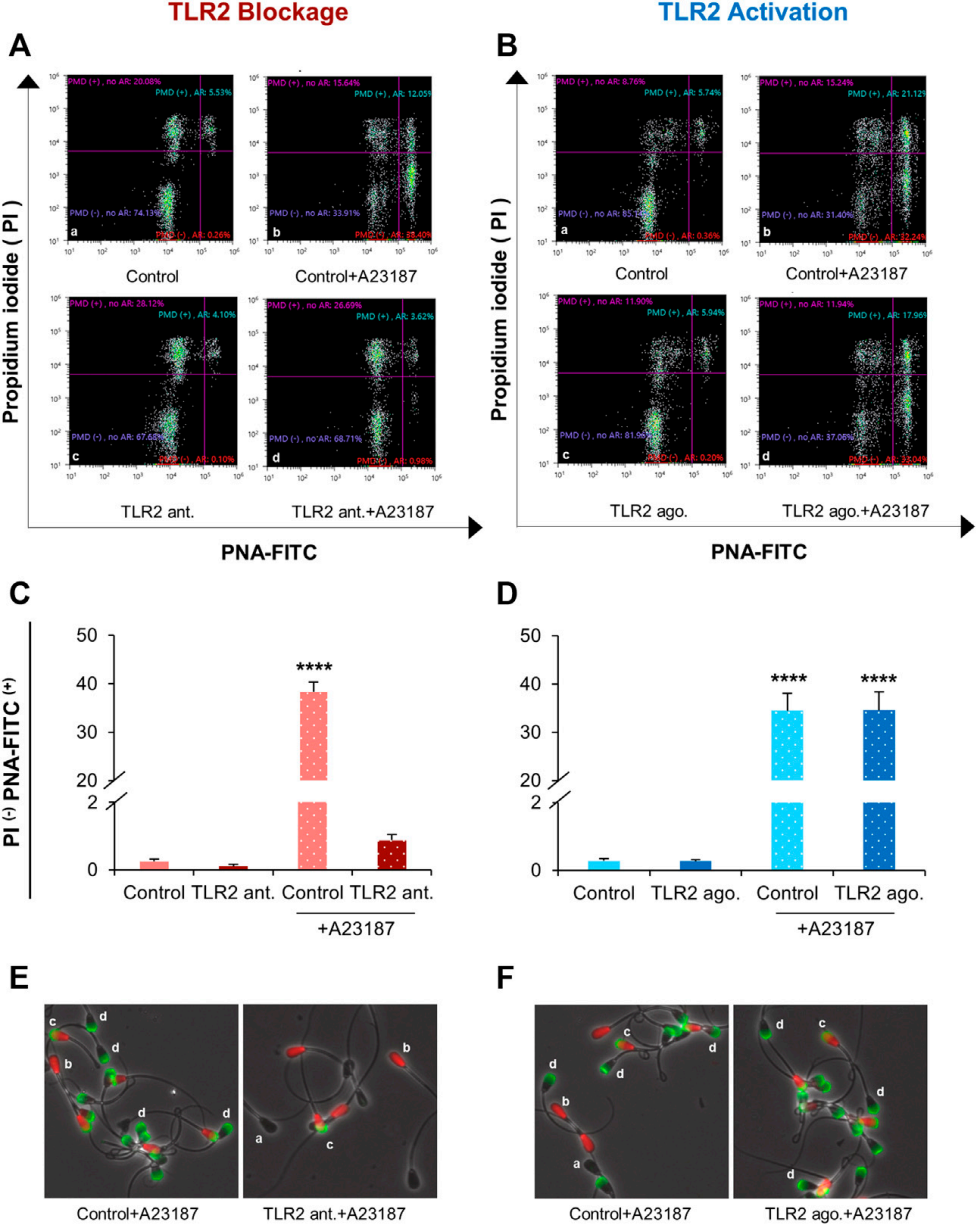
Effect of TLR2 antagonist and agonist treatment on sperm acrosome reaction (AR) triggered by A23187**.** Swim-up sperm were pre-treated by TLR2 antagonist (100 µM) or TLR2 agonist (100 ng ml^−1^) for 30 min, washed, and triggered with or without 1 µM A23187 for 60 min. Then sperm were incubated with fluorescein peanut agglutinin FITC-conjugate (PNA-FITC; for detection of induction of acrosome reaction (AR)) and propidium iodide (PI; for detection of plasma membrane damage (PMD)) for 8 min at 38.5°C in dark. The percent of live and acrosome-reacted sperm was analyzed by fluorescence microscopy and flow cytometry. Untreated sperm were kept as a negative control **(A)** Representative dot plot diagram; a: Control; c: 100 μM TLR2 antagonist; b: Control +1 μM A23187; d: 100 μM TLR2 antagonist +1 μM A23187 and **(C)** Analysis of PNA-FITC and PI staining of bovine sperm in different treatment groups by flow cytometry **(B)** Representative dot plot diagram; a: Control; c: 100 ng ml^−1^ TLR2 agonist; b: Control +1 μM A23187; d: 100 ng ml^−1^ TLR2 agonist +1 μM A23187 and **(D)** Analysis of PNA-FITC and PI staining of bovine sperm in different treatment groups by flow cytometry **(E)** Representative images of TLR2 antagonist-treated sperm or **(F)** TLR2 agonist-treated sperm stained with FITC-PNA and PI viewed with fluorescence microscope (200×). a: live intact acrosome, b: dead intact acrosome, c: dead reacted acrosome, d: live reacted acrosome. Data reported as means ± S.E.M of five independent experiments. Asterisks denote a significant variance **** (*p* < 0.0001) between the different groups compared to control.

To further confirm our hypothesis, we evaluated the impact of sperm TLR2 on the chemical induction of AR by calcium ionophore A23187. Initially, flow cytometry results showed that the addition of 1 µM of A23187 induced AR in sperm compared to control (38.33 ± 2.05% vs 0.28 ± 0.06%). However, the blockage of TLR2 did not independently interfere with sperm AR, but it suppressed A23187-triggered AR (Figure 6A,C,E). On the other hand, the activation of TLR2 interfered with sperm AR neither alone nor in combination with A23187 compared to the control or A23187-triggered AR group, respectively (Figure 6B,D,F). These findings further confirm a pivotal connection between sperm TLR2 system and their interaction with oocytes, possibly through the regulation of AR induction.

### TLR2 System Manipulates the Intracellular Calcium (Ca^2+^) Uptake in A23187-Triggered Sperm

**FIGURE 7:**
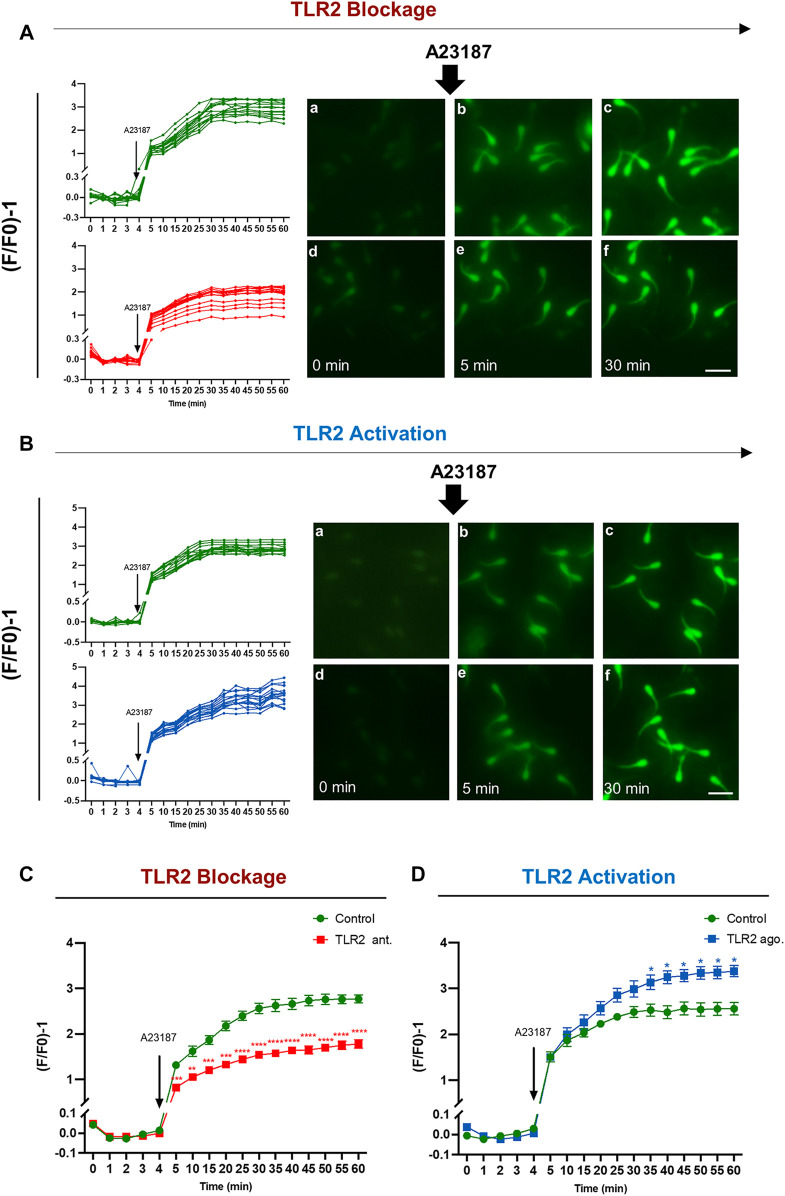
TLR2 system manipulates the intracellular calcium uptake by bovine sperm. Swim-up sperm were pre-treated by TLR2 antagonist (100 µM) or TLR2 agonist (100 ng ml^−1^) for 30 min, washed, and loaded with 5 µM Fluo-4 AM for 40 min at 38.5°C in the dark before adding calcium ionophore A23187 (1 µM as a final concentration). The sperm fluorescence was measured by the fluorescence microscope. Images were captured every 5 min for a total of 1 h, with A23187 added after the initial five readings (every 1 min). The images were analyzed using BZ-X800 Analyzer and each sperm head was selected as the region of interest. Data were normalized using the following equation (F/F0)-1. where F0 is the average of the first five readings before the addition of A23187 and F is the fluorescence intensity obtained at each time point **(A)** Single-cell imaging of Fluo-4 fluorescence in bovine sperm in response to treatment: Control (a–c) and 100 µM TLR2 antagonist (d–f) **(B)** Single-cell imaging of Fluo-4 fluorescence in bovine sperm in response to treatment: Control (a–c) and 100 ng ml^−1^ TLR2 agonist (d–f). Photos were taken before treatment (a, d), at 5 min (b, e), and 30 min (c, f) (Left) Measurement for individual sperm in each treatment for 60 min incubation. Original magnification ×200(a–f). Scale bar = 20 μm **(C)** TLR2 antagonist decreases the average intracellular calcium uptake by bovine sperm in response to treatment with A23187 **(D)** TLR2 agonist increases the average intracellular calcium uptake by bovine sperm in response to treatment with A23187. Data reported as means ± S.E.M of five independent experiments. Asterisks denote a significant variance * (*p* < 0.05); ** (*p* < 0.01); *** (*p* < 0.001); **** (*p* < 0.0001) between the treatment group compared to control.

TLR2 pathway regulates calcium (Ca^2+^) mobilization through the cell (Yu et al., 2014; Conejeros et al., 2015), which is essential for the acquisition of sperm fertilizing competence through induction of capacitation and AR (Florman and First, 1988a; Florman and First, 1988b; Parrish et al., 1999). Therefore, this experiment was conducted to identify the impact of TLR2 pathway on the intracellular Ca^2+^ uptake by bull sperm using single-cell imaging. The results showed that the exposure of sperm to TLR2 antagonist/agonist alone did not induce detectable changes in sperm Ca^2+^ influx (data not shown). Once A23187 was added, individual sperm immediately experienced the fluorescence intensity in all tested groups. In the control group, fluorescence intensity elevated sharply once A23187 was added, then showed a sustained and sluggish elevation, and finally kept stable for individual sperm. However, TLR2 antagonist (Supplementary video 2) treated sperm showed lower levels of fluorescence compared to control (Supplementary video 1). TLR2 agonist (Supplementary video 4) treated sperm showed higher levels of fluorescence compared to control (Supplementary video 3) (Figures 7A–D).

## Discussion

Accumulating evidence is building that different TLRs, as innate immune receptors, have been implicated in the regulation of several physiological functions of female reproduction including ovulation, fertilization, gestation, and parturition (Shimada et al., 2006; Liu et al., 2008; Kannaki et al., 2011). The current study sheds new light on the functional relevance of the TLR2 system in the acquisition of sperm fertilizing competence at the sperm-oocyte interface during the fertilization in bovine. Here, we adapted IVF models to investigate the impact of sperm TLR2 on sperm-oocyte interactions. Specifically, the results showed that the TLR2 system partly relates to the AR induction to penetrate ZP and fertilize oocytes, that is possibly mediated by Ca^2+^ transmembrane influx.

It was evident that stimulation of TLR2 pathway in cumulus cells of ovulated COCs indirectly stimulates sperm capacitation to enhance fertilization (Shimada et al., 2008). Also, sperm express TLR2 (Fujita et al., 2011; Akthar et al., 2020). These facts prompted us to hypothesize that the TLR2 system is directly involved in sperm interactions with oocytes for preparing sperm to fertilize the oocyte. The current results showed that the ligation of sperm TLR2 with its specific antagonist/agonist before being co-cultured with COCs down/up-regulated cleavage and blastocyst rates. Similar responses were obtained when higher (5×10^6^ ml^−1^) or lower (0.1×10^6^ ml^−1^) concentrations of sperm were used during IVF (Supplementary Figure S2). Importantly, testing our hypothesis using a wide range of sperm concentrations enabled us to exclude the possibility of biased results due to abnormal fertilization by polyspermy that might develop by using a high sperm number during IVF (Li et al., 2003; Snook et al., 2011). Therefore, we suggest that such responses were partially independent of the number of sperm assigned to fertilize oocytes but probably related to the fertilizing competence of individual sperm. Additionally, the results showed that the degree of stimulative effect by TLR2 agonist on the cleavage and blastocyst rates was relatively lower than that of suppressing effect by TLR2 antagoinst especially with high sperm concentrations (5 ×10^6^ sperm ml^−1^) in IVF (Supplementary Figure S2). This might account for the presence of endogenous ligands for the TLR2 pathway such as low molecular weight hyaluronan, obtained from the degradation of the hyaluronan-rich matrix of ovulated COCs by hyaluronidase released from sperm (Shimada et al., 2008).

Cellular mechanisms of sperm-COCs interactions for the induction of fertilization comprise three consequential levels including invasion of cumulus cell layers followed by penetration of ZP and finally, the fusion with oocytes cell membrane for transmission of the paternal genetic message encoded in the DNA (Cox et al., 1993; Anifandis et al., 2014). To identify the functional role of sperm TLR2 in the process of sperm-COCs interaction, the sperm pre-treated with TLR2 antagonist/agonist were co-cultured with cumulus-free oocytes or zona-free oocytes, and the cleavage rate was evaluated. Our data showed that the blockage or activation of the TLR2 pathway selectively manipulated the cleavage rate in cumulus-free oocytes but not in zona-free oocytes, signifying that the TLR2 pathway is involved mainly in sperm-ZP interactions, rather than the sperm-oocyte fusion. Likewise, the average numbers of ZP-bound and/or ZP-penetrated sperm were down/up-regulated by the blockage/activation of the TLR2 pathway in sperm. Sperm-ZP binding and penetration are crucial steps during fertilization (Oehninger et al., 1997) since ZP is the last barrier for the sperm before fertilizing the oocyte (Coutinho daSilva et al., 2012). Previous studies have demonstrated that lower sperm binding and penetration ratios are major causes of infertility in humans (Liu and Baker, 2000), mice (Dai et al., 2017), and bovine (Hamze et al., 2020). Therefore, it seems that the TLR2 system in bull sperm takes part in the dynamic interactions of sperm with ZP during oocyte fertilization.

Cumulus cells attract, trap, and select active sperm during the process of fertilization (Cox et al., 1993). One could argue that the current findings might be due to the direct impact of TLR2 antagonist/agonist on sperm viability and motility parameters. Especially, during pathological conditions, the drastic activation of the TLR2 pathway (using a relatively high concentration of TLR2 agonist, peptidoglycan; 1 μg ml^−1^ or Pam3Cys; 10 μg ml^−1^ for long incubation time; 6 h) reduced sperm total and progressive motility through decreasing the level of ATP production in mice (Zhu et al., 2016). In our model, the CASA analysis revealed that the pre-treatment of sperm with TLR2 antagonist or agonist for 30 min did not affect the sperm motility parameters at different time points in the subsequent culture. This suggests that the TLR2 pathway regulates sperm-ZP binding and penetration and subsequent sperm fertilizing ability without disrupting their motility and viability.

Clearly, the fertilizing spermatozoon must undergo AR to penetrate the ZP, in which the outer acrosomal membrane fuses with the overlying plasma membrane (Yanagimachi, 1994; Mao and Yang, 2013), Our results showed that the blockage/activation of the TLR2 pathway reduced/increased the AR in ZP-attached sperm. Additionally, the results showed that the blockage of TLR2 strongly reduced AR in A23187-triggered sperm. In contrast, the activation of the TLR2 pathway did not affect the high level of AR induction in A23187-triggered sperm. Also, our preliminary observation showed that increasing the concentrations of A23187 and/or TLR2 agonist did not induce a further increase in AR induction in A23187-triggered sperm (data not shown), suggesting that A23187 evoked the maximum threshold plateau of induction of AR *in vitro* which cannot be further enhanced by using any other stimulus. Together, these results suggest that the TLR2 pathway mediates sperm AR in response to ZP attachment.

TLR2 is a transmembrane receptor and its binding with a specific agonist induces the phosphorylation of several intracellular signaling adaptor proteins through the MyD88-dependent signaling pathway (Akira et al., 2001). Our recent observations showed that the treatment of sperm by TLR2 agonist or antagonist did not affect TLR2 localization and expression in sperm (Akthar et al., 2020). In mice, phosphatidylinositol 3-kinase (PI3K), as one of TLR2 signal transduction proteins (Zhu et al., 2016), is involved in the process of AR induction by ZP (Jungnickel et al., 2007). In support of this, the phosphorylation levels of PI3K were increased during the capacitation of bovine sperm (Rotman et al., 2010). Therefore, we suggest that PI3K could be the candidate to act as a commonly-shared signaling protein of the TLR2 pathway for the regulation of ZP-induced AR. Further investigations are needed to explore the downstream adaptor proteins of the TLR2 signaling pathway which are involved in regulating the process of ZP-triggered AR in bovine.

Increasing the intracellular Ca^2+^ triggers multiple physiological events in spermatozoa, such as hyperactivation, chemotaxis, capacitation, and acrosomal reaction in several mammalian species (Darszon et al., 2011; Correia et al., 2015). Acrosome serves as a store of Ca^2+^ which has been entered during capacitation and mobilized into the cytoplasm during AR (Watson and Plummer, 1986; Chiu et al., 2010). It has been demonstrated that the TLR2 pathway regulates Ca^2+^ mobilization through the cell; TLR2 agonist, Pam3Cys, stimulates Ca^2+^ influx in neutrophils (Conejeros et al., 2015), human mast cells (Yu et al., 2014), and airway epithelial cells *via* TLR2-dependent signaling and modulates proinflammatory response to bacterial infection (Chun and Prince, 2006). Therefore, we hypothesized that the TLR2 pathway regulates AR *via* mediating Ca^2+^ transmembrane transport. In support of this hypothesis, we observed that the exposure of sperm to TLR2 antagonist/agonist reduced/increased the Ca^2+^ influx into sperm during the process of AR induction using A23187. Inappropriately, we could not quantify the intracellular Ca^2+^ level of individual attached-ZP sperm because of the very limited number of those sperm mechanically detached from ZP especially after loading with Fluo-4 AM. Our observation suggests that TLR2 is involved in regulating Ca^2+^ transmembrane influx during the induction of AR, at least, in A23187-triggered sperm. However, the underlying molecular signaling of the TLR2-triggered Ca^2+^ entry mechanism and its possible link and relation with Ca^2+^ channels maily CatSper channels remains to be unclear and requires further investigations.

Overall, our findings suggest that the TLR2 system regulates sperm fertilizing competence and interactions of oocytes during the fertilization, at least *in vitro*, in cattle. The proposed mechanisms involve controlling Ca^2+^ transmembrane uptake for the induction of AR and subsequent penetration to ZP of oocytes to induce fertilization. Therefore, it seems that the sperm possibly developed a quite flexible sensing mechanism simultaneously against pathogens as well as COCs toward fertilization with the same TLR2 of the innate immune system. To the best of our knowledge, such physiological impact of sperm TLR2 on oocyte fertilization has not been described, and thus understanding its underlying mechanisms could have important translational implications in the context of assisted reproductive technology towards the improvement of fertility.

## Data Availability

The original contributions presented in the study are included in the article/Supplementary Material, further inquiries can be directed to the corresponding author.

## Supplementary Material

Supplementary Videos Representative videos for single-cell imaging of intracellular calcium uptake by bovine sperm. Swim-up sperm were pre-treated by TLR2 antagonist (0.1 % DMSO, as a control, or 100 µM) or TLR2 agonist (0.05 % ethanol, as a control, or 100 ng ml^−1^) for 30 min, washed, and loaded with 5 µM Fluo-4 AM for 40 min at 38.5°C in the dark before adding calcium ionophore A23187 (1 µM as a final concentration). The sperm fluorescence was detected by the fluorescence microscope. Images were captured every 5 min for a total of 1 h, with A23187 added after the initial 5 readings (every 1 min). Videos show time-lapse representative single-cell imaging of Fluo-4 fluorescence in bovine sperm in response to treatment: (Video 1) Control (0.1 % DMSO), (Video 2) 100 µM TLR2 antagonist; (Video 3) Control (0.05% ethanol), and (Video 4) 100 ng ml^-1^ TLR2 agonist.
