# Supplementary material for: Toll-like Receptor 2 is Involved in Calcium Influx and Acrosome Reaction to Facilitate Sperm Penetration to Oocytes During in vitro Fertilization in Cattle
Source: Front Cell Dev Biol. 2022 Feb 24;10:810961. doi: 10.3389/fcell.2022.810961 (PMC8907135; doi:10.3389/fcell.2022.810961)
Supplement: Supplementary file 4 [file DataSheet1.docx]

Supplementary Material

1. **Supplementary Figures and Tables**
   1. **Supplementary Figures**

**
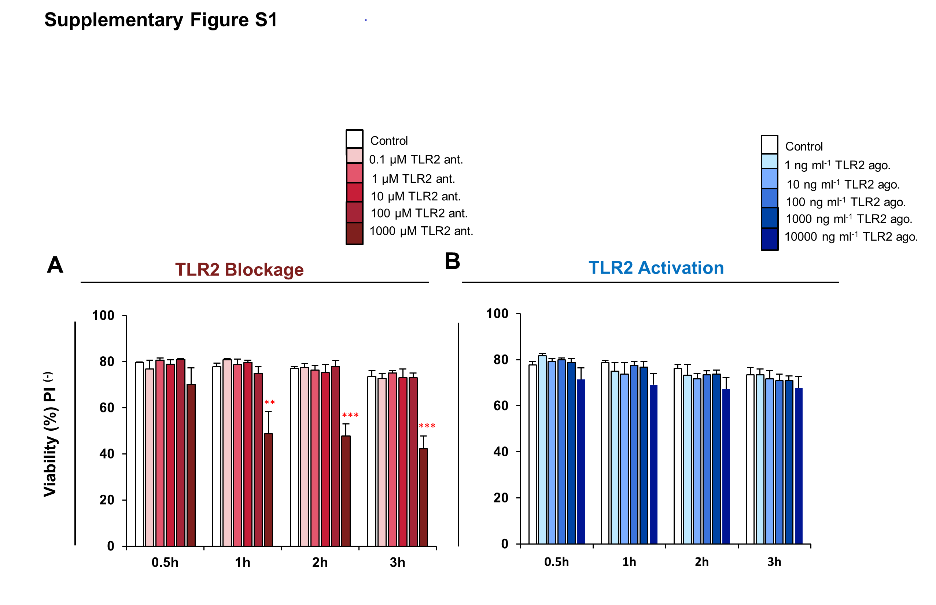
**

**Supplementary Figure S1 |** **Flow cytometrical analysis to detect the dose-dependent and time-dependent effects of (A) TLR2 antagonist or (B) TLR2 agonist on sperm viability.** Washed frozen-thawed bull sperm were treated by TLR2 antagonist (0, 0.1, 1, 10, 10^2^ and 10^3^ µM) or TLR2 agonist (0, 1, 10, 10^2^, 10^3^, and 10^4^ ng ml^−1^) for 0.5, 1, 2, and 3 h. Then, a 200 μl of sperm suspension was stained with 1 μl of 1 mg ml^−1^ propidium iodide and incubated for 8 min at 38.5°C in dark. The percentage of PI-negative (live) were determined immediately by flow cytometry. The data showed that only very high levels of TLR2 antagonist (10^3^ µM) significantly *(**P<0.01, or* ****P<0.001)* reduced sperm viability at 1, 2, or 3 h of stimulation. Data reported as means ± S.E.M of 4 independent experiments.

**
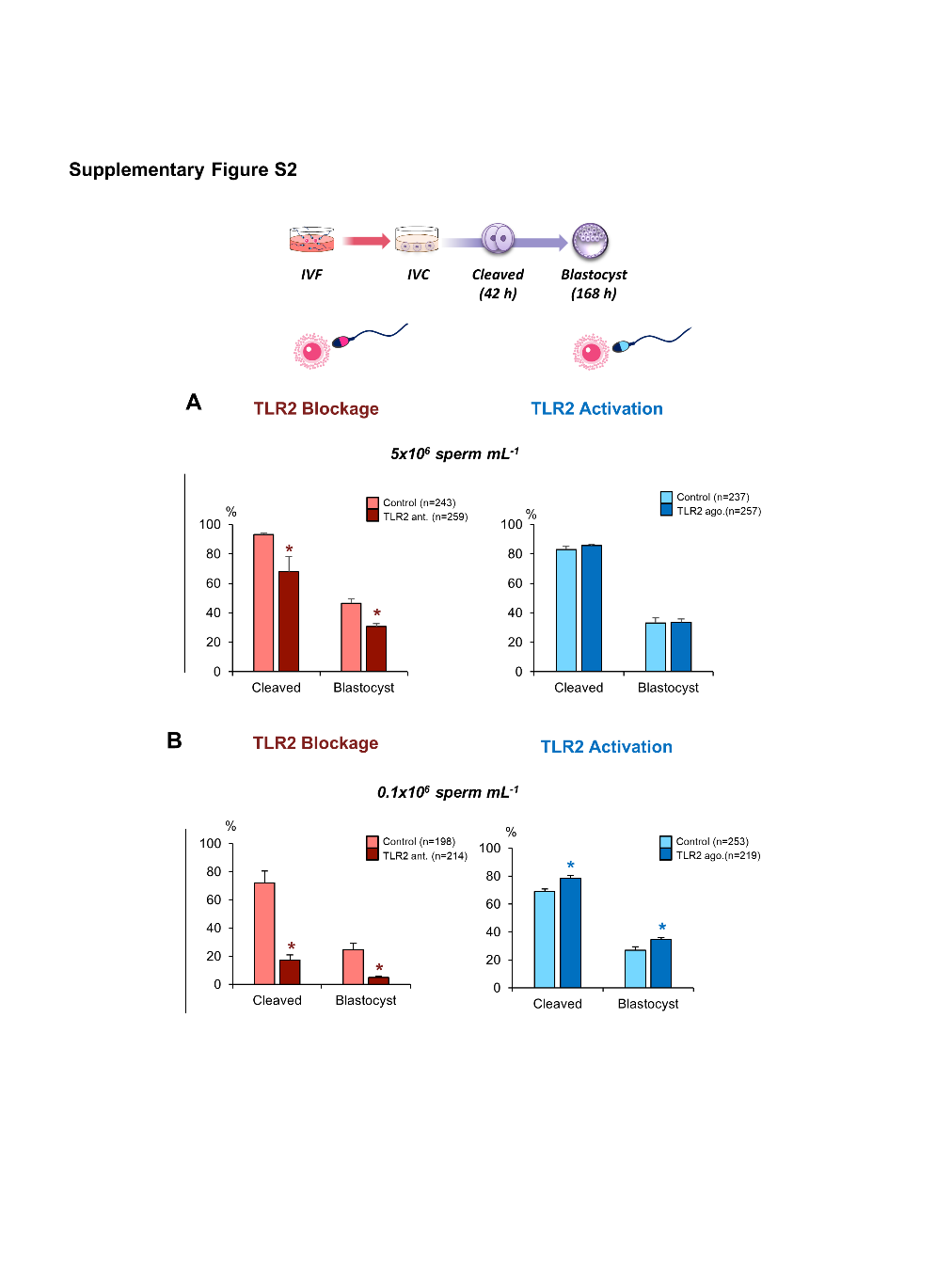
**

**Supplementary Figure S2 | Blockage/activation of sperm TLR2 suppressed/enhanced the cleavage and blastocyst rates in COCs with (A) higher (5×10^6^ sperm ml^−1^) or (B) lower (0.1×10^6^ sperm ml^−1^) concentrations of sperm during IVF model.** Sperm were pre-treated by TLR2 antagonist (100 µM) or TLR2 agonist (100 ng ml^−1^) for 30 min, washed, adjusted to a concentration of **(A)** (5×10^6^ sperm ml^−1^) or **(B)** (0.1×10^6^ sperm ml^−1^), and co-cultured with COCs for 6 h. Percentage of fertilized oocytes observed after 42 h post insemination and blastocyst on Day 7 (Day 0 = day of fertilization). Data reported as means ± S.E.M. Different superscript asterisks denote a significant difference (*p <* 0.05). The number of presumptive zygotes for each treatment group (from 3 independent experiments) is specified above each Figure.

**1.2 Supplementary Tables**

| **Supplementary Table S1 (A)\| CASA analysis to detect the dose-dependent and time-dependent effects of sperm treatment with TLR2 antagonist on sperm motility parameters.** | | | | | | | | | | |
| --- | --- | --- | --- | --- | --- | --- | --- | --- | --- | --- |
| **Time**  **(h)** | **μM** | **Total Motility（%)** | **Progressive motility**  **(%)** | **VAP**  **(µm/s)** | **VSL**  **(µm/s)** | **VCL (µm/s)** | **ALH (µm)** | **BCF**  **(Hz)** | **STR**  **(%)** | **LIN**  **(%)** |
| **0.5** | **Cont.** | 84.42±3.24 | 61.89± 1.68 | 91.95±2.25 | 79.83±1.56 | 144.33±3.64 | 2.64±0.1 | 14.82±0.06 | 78.59±1.54 | 48.17±1.18 |
|  | **0.1** | 87.33 ±0.32 | 60.79 ±3.26 | 94.85±2.36 | 82.46±2.76 | 148.32±2.76 | 2.78±0.09 | 14.38±0.31 | 76.67±1.17 | 46.98±1.51 |
|  | **1** | 89.31±2.41 | 61.25 ±1.07 | 95.94±3.46 | 83.77±4.26 | 148.58±4.41 | 2.75±0.08 | 14.52±0.26 | 77.50±1.46 | 47.06±1.75 |
|  | **10** | 89.67±1.03 | 61.40± 0.6 | 94.05±2.23 | 81.25±2.61 | 147.95±3.4 | 2.80±0.07 | 14.46±0.38 | 76.42±3.08 | 46.29±2.39 |
|  | **100** | 87.04±0.84 | 59.74 ±0.8 | 95.86±1.91 | 82.21±1.77 | 150.06±2.45 | 2.87±0.09 | 14.19±0.42 | 73.93±0.34 | 44.87±0.64 |
|  | **1000** | 65.12±7.4^*^ | 31.79± 6.32^***^ | 69.99±5.56^**^ | 57.89±6.15^***^ | 115.73±6.75^**^ | 2.46±0.09 | 11.64±0.65^***^ | 65.52±5.09 | 36.25±3.47^**^ |
| **1** | **Cont.** | 85.96±2.72 | 55.69 ±2.81 | 94.97±3.22 | 81.75±1.5 | 150.01±5.31 | 2.96±0.14 | 14.08±0.5 | 74.70±0.5 | 44.57±0.68 |
|  | **0.1** | 85.54±2.17 | 56.18 ±1.36 | 95.21±2.09 | 82.69±2.19 | 152.46±1.96 | 2.98±0.04 | 13.75±0.42 | 75.59±0.89 | 44.55±0.56 |
|  | **1** | 87.99±1.73 | 53.69 ±3.00 | 88.05±3.55 | 74.96±2.47 | 142.04±2.9 | 2.87±0.02 | 13.14±0.73 | 75.61±1.76 | 43.78±1.67 |
|  | **10** | 87.93±1.35 | 56.48 ±0.92 | 90.43±1.31 | 79.06±1.63 | 145.02±0.84 | 2.88±0.04 | 13.49±0.57 | 77.06±2.04 | 45.47±2.35 |
|  | **100** | 86.90±1.53 | 55.62 ± 1.28 | 91.08±2.83 | 77.36±3.09 | 149.20±4.86 | 3.06±0.11 | 13.29±0.54 | 75.43±0.93 | 43.43±1.46 |
|  | **1000** | 69.00±2.12^*^ | 26.14 ± 1.27^****^ | 54.65±3.98^****^ | 46.01±3.71^****^ | 95.42±5.91^***^ | 2.19±0.15^**^ | 10.81±0.21^**^ | 71.66±1.67 | 36.14±0.57^**^ |
| **2** | **Cont.** | 82.06±1.77 | 51.95± 0.79 | 96.02±0.33 | 83.02±0.12 | 154.52±1.87 | 3.14±0.07 | 13.51±0.16 | 76.02±0.5 | 44.03±1.05 |
|  | **0.1** | 81.58±2.87 | 51.81± 0.97 | 95.59±4.69 | 82.37±4.00 | 156.30±7.29 | 3.22±0.17 | 13.05±0.23 | 75.63±0.89 | 43.51±0.45 |
|  | **1** | 80.89 ±2.95 | 55.41 ±1.04 | 100.20±3.4 | 86.37±2.22 | 162.34±5.49 | 3.30±0.14 | 13.64±0.3 | 75.87±1.76 | 44.80±1.11 |
|  | **10** | 83.64 ±1.81 | 57.84± 0.79 | 99.80±3.52 | 87.30±3.54 | 160.80±3.8 | 3.29±0.03 | 13.47±0.4 | 78.75±2.04 | 46.19±0.74 |
|  | **100** | 83.48 ±1.72 | 54.25± 0.94 | 94.58±3.06 | 82.57±3.42 | 153.43±4.6 | 3.17±0.08 | 13.26±0.05 | 76.46±0.93 | 44.00±0.44 |
|  | **1000** | 54.64 ±3.02^*^ | 13.92± 4.43 | 39.22±11.05^****^ | 32.14±9.45^****^ | 69.96±17.4^***^ | 1.64±0.38^***^ | 9.89±0.34^****^ | 70.16±1.67 | 34.23±0.38^****^ |
| **3** | **Cont.** | 78.88± 1.7 | 48.04 ± 1.01 | 85.39±3.7 | 73.77±3.04 | 142.95±7.21 | 3.05±0.19 | 12.30±0.2 | 76.50±0.08 | 43.23±0.16 |
|  | **0.1** | 76.16± 1.31 | 53.86± 3.55 | 94.76±7.51 | 82.44±6.43 | 161.90±9.54 | 3.49±0.15 | 12.39±0.57 | 77.06±0.78 | 42.94±1.71 |
|  | **1** | 75.61± 1.12 | 56.06± 0.98 | 102.47±1.15 | 88.56±0.75 | 170.17±2.34 | 3.58±0.05 | 13.00±0.01 | 75.49±0.87 | 43.96±0.83 |
|  | **10** | 82.07± 3.36 | 61.52 ±2.04^**^ | 102.01±5.72 | 88.71±5.28 | 170.96±6.78 | 3.62±0.1 | 12.80±0.62 | 78.56±0.12 | 44.92±0.82 |
|  | **100** | 81.69± 1.9 | 53.64 ±1.54 | 88.44±3.62 | 76.61±3.11 | 150.91±5.73 | 3.18±0.11 | 12.14±0.11 | 76.38±1.07 | 41.78±1.01 |
|  | **1000** | 51.20± 7.07^*^ | 12.96± 5.76^****^ | 34.28±11.6^**^ | 28.38±10.16^****^ | 64.30±18.84^**^ | 1.48±0.41^**^ | 9.14±0.6^**^ | 68.04±4.21^**^ | 29.59±1.16^****^ |

| **Supplementary Table S1 (B)\| CASA analysis to detect the dose-dependent and time-dependent effects of sperm treatment with TLR2 agonist on sperm motility parameters.** | | | | | | | | | | |
| --- | --- | --- | --- | --- | --- | --- | --- | --- | --- | --- |
| **Time**  **(h)** | **ng ml^-1^** | **Total Motility（%)** | **Progressive motility**  **(%)** | **VAP**  **(µm/s)** | **VSL**  **(µm/s)** | **VCL**  **(µm/s)** | **ALH (µm)** | **BCF**  **(Hz)** | **STR**  **(%)** | **LIN**  **(%)** |
| **0.5** | **Cont.** | 88.49±2.72 | 54.98±2.9 | 92.00±3.02 | 77.66±3.91 | 146.41±3.81 | 2.88±0.08 | 13.58±0.44 | 71.03±2.66 | 42.33±2.01 |
|  | **1** | 84.72±3.12 | 51.96±2.83 | 88.08±3.15 | 74.61±4.25 | 141.71±2.25 | 2.74±0.05 | 13.44±0.83 | 72.85±2.35 | 42.44±2.48 |
|  | **10** | 85.42±1.13 | 51.16±1.9 | 87.56±4.78 | 74.49±4.41 | 140.45±6.45 | 2.70±0.08 | 13.37±0.55 | 74.13±2.09 | 43.32±1.75 |
|  | **100** | 88.63±2.76 | 57.05±3.88 | 95.70±4.41 | 82.09±4.2 | 150.77±3.48 | 2.96±0.06 | 14.05±0.67 | 75.41±1.9 | 44.94±2.39 |
|  | **1000** | 90.68±0.75 | 56.90±3.88 | 91.61±8.32 | 78.63±7.45 | 143.50±4.8 | 2.68±0.12 | 14.26±1.12 | 75.40±2.49 | 45.63±3.65 |
|  | **10000** | 78.76±2.15^*^ | 54.04±1.15 | 94.85±0.61 | 80.00±0.35 | 153.13±1.86 | 2.98±0.05 | 14.13±0.1 | 73.20±0.61 | 44.17±0.62 |
| **1** | **Cont.** | 88.43±0.76 | 55.25±0.44 | 94.09±2.31 | 80.74±1.61 | 153.09±3.47 | 3.07±0.08 | 13.19±0.33 | 74.99±1.68 | 43.05±0.82 |
|  | **1** | 86.56±2.34 | 55.86±1.95 | 99.42±3.14 | 84.99±3.04 | 158.91±6.33 | 3.17±0.14 | 13.78±0.47 | 75.27±2.21 | 44.36±2.29 |
|  | **10** | 85.45±1.22 | 55.43±1.18 | 94.91±2.54 | 81.58±1.46 | 155.18±3.4 | 3.20±0.09 | 13.18±0.42 | 74.48±1.44 | 42.90±0.78 |
|  | **100** | 84.99±0.7 | 53.81±3.21 | 92.25±4.03 | 78.09±3.32 | 152.40±7.1 | 3.11±0.14 | 12.97±0.35 | 72.71±1.19 | 41.55±1.36 |
|  | **1000** | 86.49±1.18 | 53.90±1.85 | 89.35±2.79 | 76.58±1.69 | 146.14±3.03 | 2.95±0.05 | 13.14±0.23 | 73.79±1.07 | 42.15±1.21 |
|  | **10000** | 80.28±0.99^*^ | 54.69±1.2 | 98.70±0.36 | 83.32±0.72 | 165.01±1.3 | 3.48±0.05 | 12.78±0.14 | 71.22±1.36 | 41.01±0.69 |
| **2** | **Cont.** | 81.76±0.43 | 54.56±0.64 | 96.40±0.82 | 85.30±0.71 | 158.64±1.61 | 3.27±0.06 | 12.99±0.13 | 78.08±0.35 | 44.61±0.68 |
|  | **1** | 83.76±0.97 | 55.08±1.35 | 93.28±2.23 | 81.18±2.77 | 155.91±3.19 | 3.26±0.03 | 12.62±0.38 | 77.06±1.03 | 42.65±0.39 |
|  | **10** | 82.48±2.27 | 57.64±2.47 | 96.89±4.64 | 82.92±4.05 | 162.86±4.57 | 3.43±0.07 | 12.89±0.47 | 76.62±1.3 | 43.76±2.1 |
|  | **100** | 86.59±1.6 | 58.10±2.28 | 95.05±1.41 | 81.81±1.2 | 156.36±2.59 | 3.28±0.04 | 13.11±0.08 | 77.20±0.4 | 44.44±0.39 |
|  | **1000** | 84.71±0.74 | 55.63±2.29 | 89.53±4.93 | 76.70±3.66 | 151.38±6.55 | 3.25±0.17 | 12.67±0.15 | 76.27±1.15 | 42.72±0.57 |
|  | **10000** | 71.60±4.01^*^ | 50.17±3.85 | 99.92±1.98 | 82.14±1.53 | 168.07±1.84 | 3.58±0.06^*^ | 13.12±0.37 | 70.53±0.47^***^ | 40.83±0.89 |
| **3** | **Cont.** | 80.87±1.45 | 56.78±0.91 | 95.52±4.09 | 82.46±3.91 | 158.05±4.82 | 3.35±0.07 | 12.80±0.4 | 76.65±1.25 | 44.29±1.19 |
|  | **1** | 80.40±1.15 | 58.45±1.32 | 100.26±1.78 | 88.13±1.5 | 163.92±2.49 | 3.40±0.12 | 13.11±0.31 | 78.44±1.8 | 45.71±1.03 |
|  | **10** | 78.30±1.14 | 57.69±1.71 | 101.97±5.03 | 88.23±4.86 | 167.99±5.44 | 3.47±0.06 | 13.14±0.41 | 78.18±0.76 | 45.05±0.93 |
|  | **100** | 76.34±1.12 | 51.52±1.87 | 93.93±0.47 | 81.18±0.34 | 157.46±2.28 | 3.27±0.03 | 12.63±0.08 | 75.62±1.37 | 42.66±1.05 |
|  | **1000** | 82.23±3.98 | 48.93±5.09 | 78.68±10.15 | 66.94±8.92 | 134.41±15.3 | 2.87±0.3 | 11.92±0.66 | 77.56±2.45 | 42.47±2.11 |
|  | **10000** | 69.54±2.9^*^ | 42.44±4.19^*^ | 88.20±8.51 | 70.54±9.41 | 150.28±11.56 | 3.16±0.18 | 12.62±0.6 | 65.08±3.38^**^ | 37.09±3.15^*^ |

**Supplementary Table S1 | CASA analysis to detect the dose-dependent and time-dependent effects of sperm treatment with (A) TLR2 antagonist or (B) TLR2 agonist on sperm motility parameters.** Washed frozen-thawed bull sperm (5-10×10^6^ sperm ml^-1^) were treated by TLR2 antagonist (0, 0.1, 1, 10, 100 and 1000 µM) or TLR2 agonist (0, 1, 10, 100, 1000, and 10000 ng ml^−1^) for 0.5, 1, 2, and 3 h. Then, different sperm motility parameters (total motility (%), progressive motility (%), average path velocity (VAP, µm/s), straight-line velocity (VSL, µm/s), curvilinear velocity (VCL, µm/s), amplitude of lateral head displacement (ALH, µm), beat cross frequency (BCF, Hz), straightness (STR, %), and linearity (LIN, %)) were analyzed by CASA system. The data showed that only very high levels of TLR2 antagonist (10^3^ µM) or TLR2 agonist (10^4^ ng ml^−1^) significantly *(*P<0.05, **P<0.01,* ****P<0.001, or ****P<0.0001)* reduced most of sperm motility parameters at different time points of stimulation. Data reported as means ± S.E.M of 4 independent experiments.

| **Supplementary Table S2 (A): CASA analysis to detect the effects of pre-treatment of sperm with TLR2 antagonist (100 µM) for 30 min on sperm motility parameters in the subsequent culture** | | | | | | | | |
| --- | --- | --- | --- | --- | --- | --- | --- | --- |
| **Sperm**  **Parameters** | **0 h** | |  | **1 h** | |  | **3 h** | |
|  | **Control** | **TLR2 ant.** |  | **Control** | **TLR2 ant.** |  | **Control** | **TLR2 ant.** |
| **Total Motility**  **（%）** | 84.55 ± 1.35 | 84.22 ± 2.47 |  | 73.52 ± 5.48 | 72.46 ± 6.97 |  | 70.98 ± 5.51 | 69.00 ± 5.46 |
| **Progressive Motility (%)** | 65.46 ± 2.37 | 60.76 ±1.68 |  | 54.92 ± 2.01 | 51.91 ± 1.38 |  | 43.29 ± 4.07 | 39.71 ± 3.64 |
| **VAP (µm/s)** | 111.77 ± 8.32 | 112.76 ± 4.73 |  | 98.17 ± 4.16 | 88.04 ± 9.37 |  | 92.70 ± 7.30 | 90.18 ± 6.36 |
| **VSL (µm/s)** | 99.31 ± 9.08 | 98.16 ± 4.88 |  | 84.79 ± 4.37 | 76.09 ± 8.20 |  | 81.17 ± 6.02 | 77.90 ± 5.36 |
| **VCL (µm/s)** | 166.03 ± 7.86 | 163.15 ± 4.67 |  | 152.15 ± 4.45 | 153.17 ± 5.21 |  | 137.94 ± 10.75 | 131.36 ± 8.86 |
| **ALH (µm)** | 2.98 ± 0.09 | 3.14 ± 0.06 |  | 2.99 ± 0.10 | 3.00 ± 0.14 |  | 2.67 ± 0.24 | 2.57 ± 0.22 |
| **BCF (Hz)** | 15.34 ± 1.41 | 14.69 ± 0.60 |  | 13.35 ± 0.46 | 12.81 ± 0.65 |  | 13.86 ± 0.73 | 13.47 ± 0.26 |
| **STR (%)** | 78.58 ± 4.63 | 78.49 ± 1.19 |  | 74.74 ± 3.75 | 75.94 ± 2.24 |  | 78.94 ± 2.06 | 78.71 ± 1.38 |
| **LIN (%)** | 52.95 ± 4.25 | 53.26 ± 1.34 |  | 47.95 ± 2.95 | 42.84 ± 6.00 |  | 51.49 ± 2.25 | 52.42 ± 1.17 |

| **Supplementary Table S2 (B):** **CASA analysis to detect the effects of pre-treatment of sperm with TLR2 agonist (100 ng ml^−1^)** **for 30 min** **on sperm motility parameters in the subsequent culture** | | | | | | | | |
| --- | --- | --- | --- | --- | --- | --- | --- | --- |
| **Sperm**  **Parameters** | **0 h** | |  | **1 h** | |  | **3 h** | |
|  | **Control** | **TLR2 ago.** |  | **Control** | **TLR2 ago.** |  | **Control** | **TLR2 ago.** |
| **Total Motility**  **（%）** | 88.66 ± 1.42 | 90.93 ± 0.69 |  | 89.44 ± 1.55 | 86.35 ± 1.53 |  | 83.99 ± 1.8 | 82.45 ± 1.15 |
| **Progressive Motility (%)** | 75.39 ± 2.68 | 75.40 ± 1.47 |  | 69.77 ± 4.37 | 65.20 ± 1.40 |  | 59.50 ± 3.69 | 56.42 ± 1.29 |
| **VAP (µm/s)** | 117.22 ± 3.57 | 118.64 ± 3.19 |  | 109.51 ± 5.73 | 105.43 ± 1.94 |  | 97.03 ± 4.66 | 93.89 ± 1.76 |
| **VSL (µm/s)** | 106.17 ± 3.19 | 107.84 ± 3.88 |  | 98.13 ± 4.65 | 94.98 ± 1.89 |  | 88.03 ± 4.08 | 84.90 ± 1.38 |
| **VCL (µm/s)** | 168.34 ± 2.29 | 170.68 ± 3.67 |  | 165.25 ± 8.23 | 159.14 ± 2.74 |  | 145.23 ± 8.19 | 141.12 ± 3.92 |
| **ALH (µm)** | 2.94 ± 0.07 | 3.01 ± 0.09 |  | 3.11 ± 0.13 | 2.99 ± 0.05 |  | 2.75 ± 0.17 | 2.69 ± 0.10 |
| **BCF (Hz)** | 16.52 ± 0.39 | 16.54 ± 0.78 |  | 15.61 ± 0.51 | 15.38 ± 0.40 |  | 14.84 ± 0.34 | 14.75 ± 0.27 |
| **STR (%)** | 83.55 ± 1.89 | 84.41 ± 2.12 |  | 84.42 ± 0.53 | 83.94 ± 0.60 |  | 84.53 ± 0.60 | 84.82 ± 0.99 |
| **LIN (%)** | 56.98 ± 2.06 | 57.89 ± 1.74 |  | 54.46 ± 1.00 | 54.05 ± 0.92 |  | 55.44 ± 0.82 | 56.20 ± 1.21 |

**Supplementary Table S2 |**  **CASA analysis to detect the effects of pre-treatment of sperm with (A) TLR2 antagonist (100 µM) or (B) TLR2 agonist (100 ng ml^−1^)** **for 30 min** **on sperm motility parameters in the subsequent culture.** Washed frozen-thawed bull sperm were pre-treated by TLR2 antagonist (100 µM) or TLR2 agonist (100 ng ml^−1^) for 30 min, washed, adjusted to a concentration (5-10×10^6^ sperm ml^-1^), and further cultured for 1, and 3 h. Then, different sperm motility parameters (total motility (%), progressive motility (%), average path velocity (VAP, µm/s), straight-line velocity (VSL, µm/s), curvilinear velocity (VCL, µm/s), amplitude of lateral head displacement (ALH, µm), beat cross frequency (BCF, Hz), straightness (STR, %), and linearity (LIN, %)) were analyzed by CASA system at different time points (0, 1, and 3 h). Data reported as means ± S.E.M of 5 independent experiments.

**2. Supplementary Videos**

**Supplementary Videos | Representative videos for single-cell imaging of intracellular calcium uptake by bovine sperm.** Swim-up sperm were pre-treated by TLR2 antagonist (0.1 % DMSO, as a control, or 100 µM) or TLR2 agonist (0.05 % ethanol, as a control, or 100 ng ml^−1^) for 30 min, washed, and loaded with 5 µM Fluo-4 AM for 40 min at 38.5°C in the dark before adding calcium ionophore A23187 (1 µM as a final concentration). The sperm fluorescence was detected by the fluorescence microscope. Images were captured every 5 min for a total of 1 h, with A23187 added after the initial 5 readings (every 1 min). Videos show time-lapse representative single-cell imaging of Fluo-4 fluorescence in bovine sperm in response to treatment: (Video 1) Control (0.1 % DMSO), (Video 2) 100 µM TLR2 antagonist; (Video 3) Control (0.05% ethanol), and (Video 4) 100 ng ml^-1^ TLR2 agonist.
